# Top-down Determination of Fluctuations in Topographic Measurements

**DOI:** 10.3390/ma16020473

**Published:** 2023-01-04

**Authors:** Julie Lemesle, Clement Moreau, Raphael Deltombe, François Blateyron, Joseph Martin, Maxence Bigerelle, Christopher A. Brown

**Affiliations:** 1 Valutec, Université Polytechnique Hauts-de-France, CEDEX 9, 59314 Valenciennes, France; 2 Université Polytechnique Hauts-de-France, CNRS UMR 8201—LAMIH—Laboratoire d’Automatique, de Mécanique et d’Informatique Industrielles et Humaines, 59313 Valenciennes, France; 3 Digital Surf, 16 rue Lavoisier, 25000 Besançon, France; 4 ESSILOR, Centre Innovation et Technologies CIT3, 94000 Creteil, France; 5 Surface Metrology Laboratory, Worcester Polytechnic Institute, Worcester, MA 01609, USA

**Keywords:** fluctuation, roughness, topography

## Abstract

A top-down method is presented and studied for quantifying topographic map height (*z*) fluctuations directly from measurements on surfaces of interest. Contrary to bottom-up methods used in dimensional metrology, this method does not require knowledge of transfer functions and fluctuations of an instrument. Fluctuations are considered here to be indicative of some kinds of uncertainties. Multiple (*n*), successive topographic measurements (*z* = *z*(*x*,*y*)) are made at one location without moving the measurand relative to the measurement instrument. The measured heights (*z*) at each position (*x*,*y*) are analyzed statistically. Fluctuation maps are generated from the calculated variances. Three surfaces were measured with two interferometric measuring microscopes (Bruker ContourGT™ and Zygo NewView™ 7300). These surfaces included an anisotropic, turned surface; an isotropic, sandblasted surface; and an abraded, heterogeneous, multilayer surface having different, complex, multiscale morphologies. In demonstrating the method, it was found that few non-measured points persisted for all 100 measurements at any location. The distributions of uncertainties are similar to those of certain features on topographic maps at the same locations, suggesting that topographic features can augment measurement fluctuations. This was especially observed on the abraded ophthalmic lens; a scratch divides the topographic map into two zones with different uncertainty values. The distributions of fluctuations can be non-Gaussian. Additionally, they can vary between regions within some measurements.

## 1. Introduction

In practical surface metrology, uncertainty can be problematic. Sometimes, it is impractical to estimate uncertainties bottom-up from the metrological transfer functions of instruments. Uncertainties can be estimated top-down for industry and for applied and basic research from measurement fluctuations. The essential issue is to verify on any unknown surface, using a suitable measuring apparatus, relevant settings, and appropriate measuring protocols, that the measured topographic maps *Z_M_*(*x*,*y*) converge to *Z_T_*(*x*,*y*). With the possible exception of some regular aspects of turned surfaces, the emphasis here is on the uncertainties accompanying irregular geometric aspects of topographies rather than on what might be referred to as systematic, anisotropic distortions to a surface topography. The aim is not to quantify the fidelity of topographic measurements of a known surface, but to quantify the repeatability of measurements on any surface. The standard deviation is also referred to as an error in the sense that standard deviations are calculated with respect to the mean. The mean is considered the best representation of actual surfaces, which naturally have some irregularities.

### 1.1. Surface Metrology

Surface metrology is the measurement and analysis of topographies. All surfaces have some topographic irregularities. Real surfaces are never perfectly smooth, i.e., the heights (*z*) vary spatially from position (*x*,*y*) to position on a surface. Traditional roughness parameters, including spatial means *Sa* and spatial standard deviations *Sq* (from ISO 25178-2 [1]), are calculated from *LS* surfaces that are filtered to exclude scales or spatial frequencies outside of long and short wavelength cutoffs. Measured heights (*z*) can vary with time at a single position, i.e., between successive measurements.

In topographic measurements, heights are sampled over finite zones. Within any sampling zone, there can be many heights. The sizes and shapes of these zones and actual interactions of sensing systems with topographic features within these zones are not generally known. Sampling zones probably are not rectangular, similar to the pixels that represent them on topographic maps.

The value in surface metrology is derived first from the repeatability and reproducibility of the measurements, e.g., over time. The repeatability is quantified here by temporal standard deviations, i.e., the variance or fluctuations with time at one spatial position (*x*,*y*) in the measurement. The standard deviations at each position are calculated in respect to the temporal means, which are taken as the best representations of actual surface heights at a measurement position. Therefore, standard deviations can be considered a quantification of measurement errors at a measurement spatial position.

There is also value in finding the correlations between topographies and the processing or performance variables [2], and from discriminating topographies that were processed or that performed differently. These value-producing elements, in principle, can depend on appropriate scales, geometric characterizations, measurement qualities, and statistics [3]. Understanding the repeatability of individual height measurements in real situations impacts the value in surface metrology.

The current work shows how repeated measurements at the same position can be used to determine one kind of uncertainty, which results from attempts to repeat measurements on real surfaces under actual conditions. This is a top-down method, which does not rely on special knowledge of the measurement instruments or their metrological transfer functions. Even when efforts have been made not to move a measurand with respect to a measurement apparatus, successive measurement positions can be at slightly different positions on the measurand surface. The relative movements between measurement instruments and measurands can contribute to measurement errors.

### 1.2. Philosophical Validation of Concepts for Practical Uncertainty Determination

Knowledge of a real or true surface at all scales in topography, in most practical cases, is largely a philosophical concept. All that can be known about a surface (excepting perhaps some specially constructed surfaces at certain scales) results from the interactions between a sensor and the surface at a certain time. Therefore, the topographic measurement trueness, closeness to the actual surface, is not known on most surfaces of practical interest. Reproducibility is the best measure of accuracy in practical surface metrology, and the fluctuations in measurement heights with time at each spatial position *z*(*x*,*y*) can be used as an estimation of uncertainty or a compilation of noise in topographic measurements.

Construing heights continuously and spatially at all mathematically and infinitesimally small points can only be done by curve fitting to measurements. The topographic characterization parameters in the ISO standards (e.g., 25178-2) are generally calculated from integral equations applied to primary surfaces. Primary surfaces are obtained after applying an *S* filter to eliminate the smallest scales from the measured topographies. The exact values of the spatial frequencies or wavelengths of the smallest scales are not specified, nor are the methods or rationale for their selection. This *S* filtering and the approximation of the filtered topographic data with a polynomial facilitates treating topographies as if they are everywhere known and differentiable. This treatment is required for applying integral equations to calculate the ISO characterization parameters. The problem with applying *S* filters and polynomial approximations is that the knowledge of topographies at fine scales that could be valuable is lost in order to approximate the digital measurements with a mathematical approximation for no apparent benefit. The topographic data can be treated digitally as discrete, just as it is acquired and is treated by digital computers, which digital computers perform even when discrete data are approximated by polynomials.

For this discussion, let us consider fluctuations as indicative of some kinds of uncertainties. Uncertainties *U*(*x*,*y*) are defined by Equation (1). The main problem is, then, to estimate *U*(*x*,*y*) and its source because the true actual heights, *Z_T_*(*x*,*y*), are unknown. In Equation (1), *Z_M_*(*x*,*y*) is the measured height at a position (*x*,*y*), and *Z_T_*(*x*,*y*) is the real unknown height at this position.

(1) $${U(x,y)=Z}_{T}{(x,y)\;-\;Z}_{M}\left(x,y\right)$$

Fluctuations in height measurements can vary with the position of that measurement on a surface. They can depend on the nature of the interactions of sensors with structures on the surfaces of interest. In fact, the topographic measurement of surfaces *Z_T_*(*x*,*y*) generates a height matrix *Z_M_*(*x*,*y*) as a result of the interactions between real surfaces and a measurement instrument’s transfer function. Thereafter, all calculations (filters, shape, parameters, uncertainties) use a *Z_M_*(*x*,*y*) matrix. The topographic measurement and subsequent analyses and characterizations are independent processes.

The function *U*(*x*,*y*) is a quantification of uncertainties. It contains two components: one deterministic and one stochastic. A deterministic uncertainty assessment requires the device-specific identification of *I* parameters, *p_i_*, that result in repeatable deviations *D_i_*(*x*,*y*), which represents the accuracy of the measurement by the metrological system. Fluctuations in repeatable deviations, noted as *F_i_*(*x*,*y*), therefore generate fluctuations in *U_i_*(*x*,*y*) (Equation (2)). Global uncertainty *U*(*x*,*y*), under independent between *U_i_*(*x*,*y*), can be expressed by Equation (3). $D=\sum_{i}D_{i}\left(x,y\right)$ can only be estimated by metrological experiments on a measuring instrument and cannot be accessed by a simple measurement of *Z_M_*(*x*,*y*). Conversely, the total fluctuation, $F=\sum_{i}F_{i}\left(x,y\right)$ (random uncertainties), will generate a fluctuation on *Z_M_*(*x*,*y*) in each measurement of the surface.

(2) $$U_{i}(x,y)=D_{i}{(x,y)+F}_{i}\left(x,y\right)$$

(3) $$U(x,y)=\sum_{i}U_{i}\left(x,y\right)=\sum_{i}D_{i}\left(x,y\right)+\sum_{i}F_{i}\left(x,y\right)$$

The paradigm in surface metrology is to decompose macroscopic uncertainty *U*(*x*,*y*) into a mesoscopic system, such as ${U(x,y)=f(u}_{1}{,u}_{2}{,\ldots ,u}_{n})$, where *f* is the macroscopic model of uncertainty, and *u_i_* are uncertainties (*u_i_* values are not always independent and not necessarily dependent on their location (*x, y*)). Each component *u_i_* can itself be decomposed into a microscopic system, such as $u_{i}=g(v_{1},v_{2},\ldots ,v_{n})$, representing the interaction between the real surface and the transfer function of the instrument.

**Figure 1:**
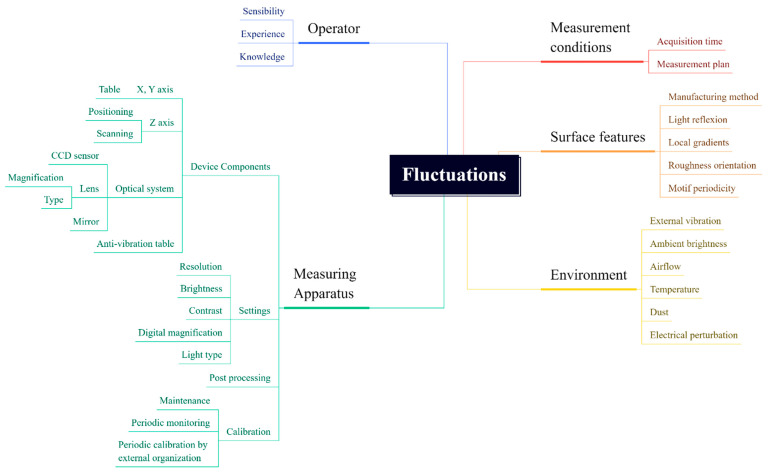
Sources of topographical fluctuations.

With just a series of measurements that can be applied wherever topographic measurements are made, this work provides a tool for determining height measurement fluctuations at scales, i.e., spatial wavelengths or frequencies, commonly studied in surface metrology. These fluctuations are due to any and all sources, such as drift or vibrations (Figure 1). This addresses a central issue for surface metrology. Basic height measurements precede whatever characterizations are used to interpret the measurements. An important part of the practical value of this work for research, manufacturing, and commerce is the ability to calculate fluctuations under actual conditions. The tool provided here can be applied regardless of filtering.

The validation of the determination of real fluctuations by this method is based on many repetitions of topographic measurements at multiple locations, each of which contains millions of measured heights made under actual, practical conditions. This is a validation of real fluctuations because there are many transient factors that influence fluctuations. Situation specific fluctuations cannot be predicted from bottom-up studies of an instrument using step heights under carefully controlled conditions. This determination of real fluctuations, which results from influences that are specific to local conditions, is naturally incorporated into the fluctuations at the time of measurement. Because this is top-down, it is not intended to qualify any particular, individual location, position, instrument, or condition with an indication trueness.

### 1.3. State-of-the-Art

The origin of uncertainties in optical measurements is described by Leach and Giusca [4] and introduced in the standard ISO 25178:600 [5]. In 2021, Leach et al. [6] published an exhaustive review on the calibration of surface topography instruments in a generic formulation and gave indications on the forthcoming standard ISO 25178:700. In their paper, they made the point that *u_i_* or *v_j_* can be independent of surface materials and topographies, so that the properties of the instrument and its environment can be determined and used in an uncertainty estimation independent of the measured topographies.

In contrast, this current paper assumes that Uncertainties are Topographically and Material Dependent, called UTMD, i.e., uncertainties depend on the material properties and complexities of topographies [7]. For this reason, some surfaces are manufactured to be closer to industrial topographies and, therefore, more appropriate for estimating practical UTMD. Areal standard artifacts were designed by NPL [8] for areal topography measurements [9]. These artifacts were 100 mm² and composed of ten multifunction silicon artifacts mounted on precision glass substrates. Eifler et al. also manufactured, by direct laser writing, acrylate-based negative-tone photo-resist standard artifacts composed of six target geometries [10]. All of these are manufactured to give different topographies on homogeneous materials with high durability.

However, UTMD is material dependent. The greatest complexity may happen when topographies and local material compositions are intimately linked together. In fact, actual surfaces are often spatially heterogeneous [11]. Topographies are often created by complex physical mechanisms, such as oxidation [12], wear [13], molding [14], cutting [15], abrasion [16], plastic deformation [17], and polymer relaxation [18]. The peaks and valleys can have different properties: optical [19] and mechanical [20]. Heterogeneity can also be material dependent due to the precipitates present in material matrices, leading to different refractive indices [21] or mechanical characteristics [22]. These could also introduce uncertainties in the height measurement. Finally, measured surface topographies can have fractal characteristics [23] with different characteristic lengths [24]. The quantification of *U*(*x*,*y*) by fluctuation propagation on heterogeneous surfaces or UTMD surfaces would present a challenge. As pointed out by Leach et al. [6], how the UTMD determination *“should be quantified and used in uncertainty estimation are still open research questions”*.

The determination of uncertainties *U*(*x*,*y*) in topographic measurements is often formalized by a bottom-up type approach. Mesoscopic uncertainties (*u_1_, u_2_, …, u_n_*), associated or not associated to microscopic uncertainties, are quantified with their influence (temperature, vibration, and material heterogeneity, for example) and introduced in an *f* model, where *f* is the final macroscopic uncertainty. For this reason, the bottom-up propagation of uncertainties facilitates the calculations of *f* [25]. The bottom-up approach [26] elucidates the origins of uncertainties and their influence on the results. This can be used to improve instruments and procedures. Bottom-up is a thorough metrological approach, which lists contributors to uncertainty as exhaustively as possible and classifies them according to their influence.

However, writing an *f* function for a UTMD remains complicated and can leave uncertainty in the propagation of uncertainties. The utility of the top-down approach is, therefore, conceivable [22]. This approach involves approximating uncertainties based on the measurement of topographies alone without the quantification of dissociated, physics-based fluctuations [27,28]. Top-down is a statistics-based phenomenological approach. With it, metrologists can quantify the variations due to a measuring system’s response to a measurement environment. In fact, it is similar to a quality map used in focus variation; the operator looks at the metrological response of the apparatus according to the settings [29]. However, the influence of topographies on quality maps is not displayed; although, it could be valuable for an operator [30].

Both approaches, bottom-up and top-down, could be used together. For example, to estimate the measurement noise, Maculotti et al. measured a surface *n* times to deduce the statistical parameters of this noise and thus isolate a *u_i_* component of the global model [31]. These approaches are complementary. In the current paper, a top-down approach is presented for a phenomenological study using statistics to quantify UTMD-type uncertainties by relating them to measured topographies.

Uncertainty can be considered a kind of noise. Noise in surface metrology has been considered something “added to the output signal occurring during the normal use of the instrument” [31]. This is in the context of measuring optical flats, essentially known surfaces. In measuring surfaces with irregular geometric components, this might not be an apt definition of noise. Here, noise is uncertainty in the repeatability of a height measurement at a position.

### 1.4. Practical Interests in Fluctuation Analysis

1. Metrological control: A metrological measuring instrument can be damaged or can degrade over time. Therefore, it is necessary to regularly monitor an instrument’s performance, i.e., to verify its measuring capability for quality assurance. The usual practice is to select reference standards certified by an accredited body. Two kinds of standards can be used: stochastic standards, in which case roughness parameters are certified (*Sa*, *Sz*, etc.); and deterministic standards (step, periodic surface, etc.), in which case deviations from an accredited geometry are certified. In neither of these two surface classes is the uncertainty of the discretized heights analyzed. In fact, all morphological indicators average this local uncertainty. The initial aim is to warn of a measurement drift in relation to the metrological reference. However, this averaging may not provide indicators capable of detecting this drift, whereas some indicators could detect it on the temporal analysis of the map *Z_M_*(*x*,*y*,*t*) and thus become a complementary measure of the measurement capability.
2. Environmental disturbance: An interest of the analysis of *Z_M_*(*x*,*y*,*t*) in conjunction with a record of environmental conditions *C_i_(t)* is to analyze the effect of the condition *i*, i.e., the influence of the environmental variables (temperature, vibration, relaxation, convection phenomena) by time inter-correlation analysis using classical time series analysis tools. This fluctuation *Z_M_*(*x*,*y*,*t*) can be material dependent under the disturbance *C_i_(t)*.
3. New recorded device optimization: The analysis of *Z_M_*(*x*,*y*,*t*) is insightful when implementing new dimensional metrology by quantifying the dimensional stability of the new device.
4. Topographic apparatus reliability. The analysis of *Z_M_*(*x*,*y*,*t*) over long periods of time (from one week to one month) allows the analysis of the long-term stability of the measurement device. In addition, environmental effects can be detected (periodic phenomena).
5. Selection of measurement instruments: With different instruments, fluctuation can differ. Fluctuation tendencies at the location where they will be used, on actual surfaces of interest, can be a factor in selection.
6. Optimization of measurement conditions: Many settings are available for topographic measurements. From an unknown topography, the settings are often obtained empirically, and often the appreciation is visual and based on the map *Z_M_*(*x*,*y*,*t*). The knowledge of *Z_M_*(*x*,*y*,*t*), by constructing adequate indicators, can provide quantitative indicators to guide the choice of optimal setting conditions.
7. Correlation with surface functionality: Functional correlations relate topographies through their measurement and characterizations, and their performance or processing. Introducing metrological fluctuations will make functionality measurements more reliable. Fluctuations can be integrated into the functionality determination (ASME B46.1 2019, Appendix A [2]).
8. Robustness of topographic characterization parameters: The sensitivity of topographic characterization parameters to fluctuations can be quantified as well. Fluctuations in *Z_M_*(*x*,*y*,*t*) can lead to large deviations in certain topographic parameters *q_i_* that are sensitive to extreme values (*Sz*, *Sp*, *Sv*) or segmentation. This effect could be quantified by studying *q_i_*(*Z_M_*(*x*,*y*,*t*)) as a function of the time for a given type of topography.
9. Optimization of topographic characterization: Height fluctuations can be integrated into the calculations of topographic characterization parameters. In response to the physical aspects of surface topography, the amplitude of the *Z_M_*(*x*,*y*,*t*) map fluctuation can depend on the position (*x*,*y*). Spatial variation in fluctuations can be considered statistically as heteroscedastic data. Often, to obtain more robust estimators, it may be customary to use indicators weighted by this variability. For example, *Sa* will integrate more weakly in its summation amplitudes having a higher variability. It is often customary to determine the uncertainties of the parameters on a map *Z_M_*(*x*,*y*,*t*) either by propagation of uncertainties or by statistics. However, the calculation of the parameter remains unchanged. The question fundamentally arises over whether the estimation of parameters should integrate this source of variability. There is a basic problem of what a topographic characterization or roughness parameter is and its statistical estimation. *Sa*, *Sz*, *Sq* are only estimators of the dispersion of a statistical distribution of heights. This estimator is required to be unbiased, efficient, convergent, and robust. An obvious bias concerns the slope parameter *Sdq* measured in interferometry. Greater slopes result in greater fluctuations or uncertainties. If the calculation is weighted, the *Sdq* will drop. However, if this averaged *Sdq* makes it possible to more robustly differentiate two surfaces because of different average *Sdq* values, then *Sdq* is more suitable for characterizing the two surfaces.
10. Outlier removal: In topographic measurements, some kinds of outliers are not repeatable to the same degree as other measured heights. Fluctuations can be an indication of doubtful points, i.e., outliers.
11. Diminishing NaNs: Certain measuring devices (interferometer) can generate, at certain positions (*x*,*y*) at a time *t_i_*, an unmeasurable point *Z*_*M*_(*x*,*y*,*t*_*i*_). Measuring several times at the same position can, in certain cases, result in a few well measurement points, providing a combined map containing fewer NaNs.

## 2. Measurement Indexing

In general, measurements of topographies at different locations on a measurand are measured many times each to produce sets of maps *M* to analyze change. *N_n_* topographic measurements are performed on surfaces *S_s_*, at moments *T_t_*, and at locations *X_x_* with a measuring instrument *A_a_* set up in a condition *C_c_* with protocol *O_o_* and post-processing *P_p_* to provide multi-maps *M* (Equation (4)).

(4) $${M,\;M}_{n}{(S}_{s}{,\;T}_{t}{,\;X}_{x}{,\;A}_{a}{,\;C}_{c}{,\;O}_{o}{,\;P}_{p}{,\;N}_{n}),\;i\;\in \;\left\{1,2,\ldots {,N}_{n}\right\}$$

Several modalities can be applied to ***M***, which will be noted and defined generally by place-holding empty squares between curly brackets, □={□,□}. For example, ${T=\{t}_{1}{,\;t}_{2}\}$ means that two maps are measured for multi-maps ***M***, created at times $t_{1}\;$ and $t_{2}$. In the case of two successive modalities ending in a single multi-map, the notation is □={□⊗□}. Therefore, ${O=\{O}_{1}\;⊗{\;O}_{2}\}$ means that the protocol $O_{1}$ is performed followed by the protocol $O_{2}$. {□} is noted if the modality is not significant for the study, and {*ϕ*} if there is no modality. The number of repetitions will be noted as exponent {*□^n^*} for non-important repetition. In the absence of ambiguity, {□} and {*ϕ*} can be omitted in Equation (4).

The following example demonstrates this definition. Pieces with certain types of finishes obtained by a specific finishing process (*tool*) are measured one hundred times at five random locations with an interferometer, *Contour GT*, with a 20× lens (Equations (5)). Simplifying, Equations (5) becomes Equations (6).

(5) $$M_{n}(S=\{tool\},\;T=\{□\},\;X=\{{□}^{5}\},\;A=\{ContourGT\},\;C=\{20x\},\;O=\{\phi \},\;P=\{\phi \},\;N=\{100\})$$

(6) $$M_{n}(S=\{tool\},\;x=\{{□}^{5}\},\;A=\{ContourGT\},\;C=\{20x\},\;N=\{100\})$$

## 3. Materials and Texturing Methods

**Figure 2:**
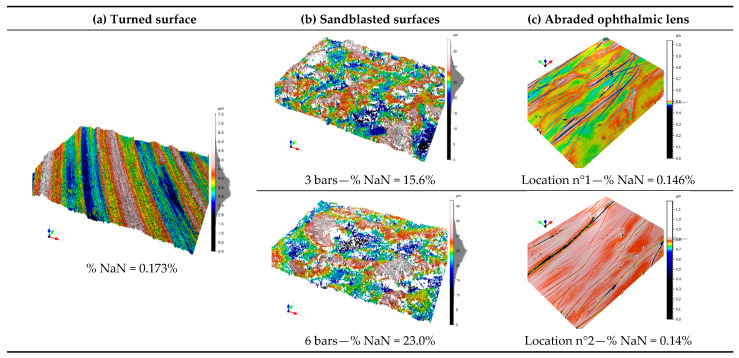
Renderings of measured topographies from surfaces turned (**a**), sandblasted under a pressure of 3 and 6 bars (**b**), and two zones of the abraded ophthalmic lens (**c**).

Three different finishes, one turned, two sandblasted, and one ophthalmic lens, are shown in Figure 2. The pieces with turned and sandblasted surfaces are made of aluminum alloy AU4G. The two sandblasted surfaces are obtained by blasting with corundum at 3 and 6 bars for 60 s. Turning was done at 120 m/min with a tungsten carbide D-type insert. Finally, the organic, biplane, and multilayered ophthalmic lens was provided by Essilor. The lens was abraded according to the Bayer test, i.e., by a back-and-forth movement of sand during 300 cycles at 150 cycles per minute [32].

**Figure 3:**
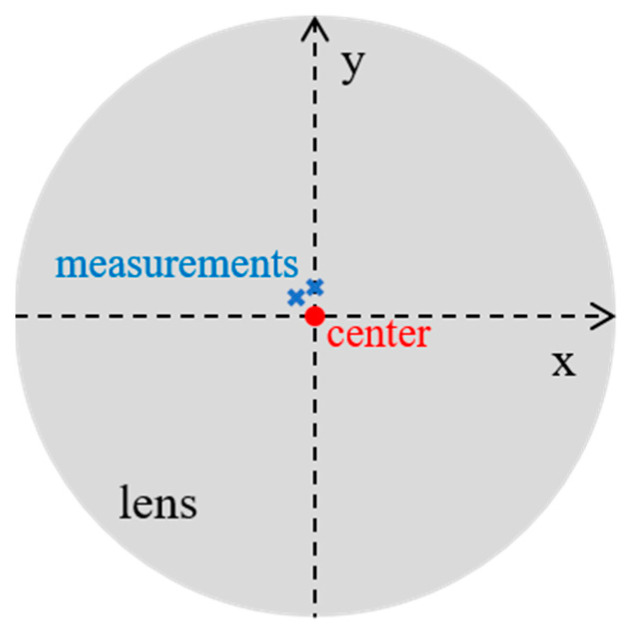
Locations of the two measured regions on the biplane ophthalmic lens.

One region is measured per process surface (turning and sandblasting) with White Light Interferometry (WLI—Bruker ContourGT™) with a 20× lens. For the ophthalmic lens, two regions were measured with Scanning White Light Interferometry (SWLI—Zygo NewView™ 7300) with a 50× lens (Figure 3). Each measurement instrument was placed on an anti-vibration table in an air-conditioned room at 20 °C. One hundred measurements, always at the same position, were performed for each set of maps *M*, which corresponds to approximately 20 min per measured region at one location. The sizes of the measured regions are 315 × 236 µm² (640 × 480 pixels) for the Bruker Contour GT™ and 140 × 105 µm² (640 × 480 pixels) for the Zygo NewView™ 7300.

## 4. Fluctuations Plotting Method

### 4.1. Mean and Variance Maps

This method of estimating fluctuations in a topographic measurement is illustrated as described above by Equation (6). Let *z*_*i*,*j*,*n*_ be the surface amplitude at the (*i, j*) coordinate point of the *n^th^* map (*n* = (1, …, *N*)) of size (*I, J*). The idea is to calculate a statistic F representing the dispersion in *M* at coordinates (*i, j*) of all maps *M_n_* (Equation (7)).

(7) $$F{(Z}_{i,j,n})=F_{i,j}$$

Importantly, there is no form removal for each map *n*. Indeed, this would introduce an influence of variation of the average level at each point *z*_*i*,*j*,*n*_ because the polynomial *Π_n_* used to remove form would introduce a difference $Z_{i,j,n}{\;-\;\Pi }_{i,j,n}$. As a consequence, all maps are reduced by the difference $Z_{i,j,N}{\;-\;\Pi }_{i,j,N}$, where *Π*_*i*,*j*,*N*_ is the plane computed from the *N* maps. This tilts the entire set of *N* maps with a constant inclination. A mean map of the *n* elementary maps, noted F=μ, is calculated first. This enables a more robust estimation of the surface amplitude while minimizing their influence on an elementary map. The mean map *μ*_*i*,*j*_ is described by Equation (8), where *ϕ* represents the non-measured data (NaN).

(8) $$\mu_{i,j}=\frac{\sum_{n=1}^{N}Z_{i,j,n}\delta_{i,j,n}}{\sum_{n=1}^{N}\delta_{i,j,n}}{\;\mathrm{and}\;\mu }_{i,j}=\phi \;\mathrm{if}\;\overset{N}{\underset{n=1}{\sum }}\delta_{i,j,n}=0\;{\mathrm{with}\;\delta }_{i,j,n}=\left\{\begin{array}{c} {1\;\mathrm{if}\;Z}_{i,j,n\;}\neq \;\phi \\ 0\;\mathrm{else} \end{array}\right.$$

Variance is expressed by the standard deviation of the heights *σ*_*i*,*j*_ at any point *z*_*i*,*j*,*n*_ of the multi-map (Equation (9)). If there are less than two points measured with *z*_*i*,*j*,*n*_, then *σ*_*i*,*j*_ is considered equal to *ϕ*.

(9) $$\sigma_{i,j}=\sqrt{\frac{\sum_{n=1}^{N}{\left(Z_{i,j,n}{-\mu }_{i,j}\right)}^{2}\delta_{i,j,n}}{\sum_{n=1}^{N}\delta_{i,j,n}}}{\;\mathrm{and}\;\sigma }_{i,j}=\phi \;\mathrm{if}\;\overset{N}{\underset{n=1}{\sum }}\delta_{i,j,n}\;<\;2$$

### 4.2. Reference for the Determination of Fluctuations

Here, fluctuations are quantified directly on measured topographies. The standard deviation *σ*_*i*,*j*_ is the fluctuation of each position or pixel height on the mean map *μ*_*i*,*j*_. This fluctuation can be compared with the total RMS roughness *S*_*q*,*n*_, *n* = (1, …, *N*). *S*_*q*,*n*_ is a standard parameter for characterizing measured topographies. Following ISO 25178-1 [33], when roughness is characterized, the measured topography can be fitted with a polynomial type F-operator *Π_d_*, where *d* is the polynomial degree. Therefore, to calculate the series of *S*_*q*,*n*_ (*S*_*q*,1_, …, *S*_*q*,*N*_), the form is removed from each measurement map *n*, and the parameter *S_q_* is calculated (Equation (10)). *S_q_* is calculated by averaging all the heights in a measurement,

(10) $$S_{q}=1Nn=1NS_{q},n\;\mathrm{with}\;Sq,n=i=1Ij=1Jzi,j,n-\;\Pi d,I,j,n2\delta i,j,ni=1Ij=1J\delta i,j,nS_{q}=\frac{1}{N}\sum_{n=1}^{N}S_{q,n}{\;\mathrm{with}\;S}_{q,n}=\;\sqrt{\frac{\sum_{i=1}^{I}\sum_{j=1}^{J}{\left(I{-\Pi }_{I,i,j,n}\right)}^{2}I}{\sum_{i=1}^{I}I}}$$

where *Π*_*d*,*i*,*j*,*n*_ is the polynomial of degree *d*, which is the best least squares interpolation of the heights *z*_*i*,*j*,*n*_. In this paper, *d* = 1 is retained.

In calculating the fluctuations *S*_*q*,*n*_, it is assumed that they are independent of each other. In mathematical terms, a correlation function taken two-by-two is null (Equation (11)).

(11) $$\mathrm{cov}\left({Sq}_{n}{,Sq}_{m}\right)=0,\;\forall \left(n,m\right),\;n\;\neq \;m\;\mathrm{Assumption}\;n{}^{\circ}1\;$$

From the assumption n°1, Equation (9) can be normalized (Equation (12)).

(12) $${\hat{\sigma }}_{i,j}=\frac{\sigma_{i,j}}{Sq}$$

This is the equation of the additional topographic fluctuation to evaluate a noise-to-signal ratio. If ${\hat{\sigma }}_{i,j}\;\ll \;1$, then fluctuations are negligible, while if ${\hat{\sigma }}_{i,j}\;\gg \;1,\;$ then the map is not interpretable. If ${\hat{\sigma }}_{i,j}=1$, measurement fluctuations are of the same order of magnitude as the measurement itself.

### 4.3. Autocorrelation of Fluctuations

The assumption n°1 independence can facilitate simplification, but it ignores temporal fluctuations. At the limit, outside any variability of the estimators built for estimating Equation (10), this assumption has strong metrological and morphological implications. In fact, this is necessary to reformulate Equation (10) similar to Gomez et al. [34]. Rather than reformulating a variance expression of fluctuations sequentially measured, they consider the variance of fluctuations on each map as a time function by introducing the acquisition frequencies *Δt* between two successive measurements *z*_*i*,*j*,*n*_ and *z*_*i*,*j*,*n*+1_. This approach offers the undeniable advantage of introducing a characteristic time to the quantification of the *n* measurement fluctuations rather than an ordered sequence, giving Equation (13).

(13) $$\mathrm{cov}\left({Sq}_{n\Delta t\;}{,Sq}_{\left(n+1\right)\Delta t}\right)=0,\;\forall n$$

The choice of *Δt* is complicated and is composed of three different times (Equation (14)). The total measuring time is, therefore, t=NΔt. *Δt_intra_* is the time necessary to measure the topography *z*_*i*,*j*,*n*_. *Δt_inter-intra_* is the intermediate time between two measurements to process and transfer data and to locate the system when stitching is used. Finally, *Δt_inter_* is the time, voluntarily chosen, of waiting between two measurements (it can be zero).

(14) $$\Delta t=\Delta t_{intra}+\Delta t_{inter\;}+\Delta t_{intra-inter}$$

In principle, *Δt_intra_* depends, at most, only slightly on *n*. This can vary depending on the measurement apparatus and function of the apparatus settings especially in the case of an apparatus with lateral scanning and stitching, which is not studied here. Many optical instruments, e.g., interferometer [35], focus variation [36,37], often perform *k* horizontal scans, which introduces a time *Δt_z_*, such as $\;\Delta t_{z}=\overset{k}{\underset{i=1}{\sum }}\Delta t_{z_{i}}$. Except for iterative algorithms that could be found in focus variation instruments, $\Delta t_{z_{i}}\;$ does not depend on height; therefore, $\Delta t_{z_{i}}=\Delta t_{z}$ and $\Delta t_{z}=k\Delta t_{z}$. Δ*t*_*z*_ is considered as an elementary time, introducing the second assumption described by Equation (15), where *z*_*i*,*j*,*n*,*k*_ is the height *z*_*i*,*j*_ of the pixel (*i, j*) from the *k*^th^ scanning plane of the *n*^th^ topographic map.

(15) $$\mathrm{cov}\left(z_{i,j,n,k}-\mu_{i,j,k}{,\;z}_{i,j,n,k+1}-\mu_{i,j,k+1}\right)=0,\;\forall \left(i,j,n,k\right)\;\mathrm{Assumption}\;n{}^{\circ}2$$

This assumption means that fluctuations are not correlated between two scanning planes of a topography. This assumption seems weak (except for stitching) because there is little chance that an external disturbance, such as vibration [38] and temperature variation [39], has a repercussion under these high frequencies; however, it can be considered as moderate in the internal case (hysteresis of the z-displacement motor [40]) or strong for some topography processing algorithms, particularly in focus variation [41]. Indeed, for this last case, it is obvious that the calculation of the focus plane height depends on the adjacent plane(s) and local topographic slopes, which depend on heights, *z*. Moreover, a diffusion of fluctuations will be introduced by the contrast function thus correlating with noise in the (*x, y*) plane. These observations lead to a third assumption (Equation (16)), contrasted with Equation (15).

(16) $$\exists \left(i,j,i’,j’\right),\;\mathrm{var}\left({\hat{\sigma }}_{i,j}\right)\;\neq \;\mathrm{var}\left({\hat{\sigma }}_{i’,j’}\right),\;i\neq i’,\;j\neq j’\;\mathrm{Assumption}\;n{}^{\circ}3$$

This third assumption is the basis of a method presented in this paper. This states in simpler terms that the fluctuations are correlated to the *z*-scanning plane due to the fact that the detections of the topography, the real unknown topographic surface, *z*_*x*,*y*_, depend on the local topography conditions in (*x, y*), which are strongly influenced by the slopes between two successive scanning heights at the focus height *z_f_* that are *dz* apart, i.e., $z_{x,y,t,z}{\;-\;z}_{x,y,t,z+dz}$. Equation (17) is obtained without formalizing the notion of gradient,

(17) $$\mathrm{var}\left(z_{{x,y,t,z}_{f}}\right)=F\left(z_{{x,y,t,z}_{f}}{\;-\;z}_{{x,y,t,z}_{f}+dz}\right)$$

where *F* is a function to be defined, which also depends on measuring the conditions and nature of the material.

Let us take the tactile profilometry with a stylus of radius curvature *r* as an example. This technique is more easily understandable and interpretable than optical topographic measurements to illustrate the previous paragraphs because they are limited to a 2D approach. A standard surface representing a step is studied. This measure is commonly performed to verify the ability of the measuring apparatus to measure the value *Δz* (Equation (18)) indicated by a reference standard [42].

(18) $$\Delta {z=z}_{x\_top}{+z}_{x\_bottom}$$

In fact, near step transitions at *x_jump_*, there is smoothing of the profile due to stylus radii [43,44], and there is a risk of jumping due to the kinetic energy of the moving stylii [45]. This implies that Equation (18) is not correct at locations where steps must be measured. Equation (19) is practically used to measure the step *Δz*. There is a correlation of fluctuations near jumps, which can be seen as two measuring planes differing in height by *Δz* and a shift of the curve in $\;\Delta {x=x}_{jump}\;-\;r$.

(19) $$\Delta {z=z}_{x,\;x\;\ll {\;x}_{jump}\;-\;r}\;{-\;z}_{x,\;x\;\gg {\;x}_{jump\;}+r}$$

Now, let us suppose that the topography of a wafer must be measured with an interferometer. Consider it flat and smooth, a perfect mathematical plane; then, only measurement noise independent of the topography is recorded. Leach et al. showed that, in this case, by subtracting two consecutive maps [34] measured at a short interval, a decorrelated noise results, and therefore, Equation (15) is valid. However, if a slight tilt is made (2° and 4°), the noise becomes strongly correlated between pixels. Whether for tactile measurements or interferometry, fluctuations depend in a general way on the local surface gradient, such that the more gradients increase, the greater the fluctuations become.

In this paper, the fluctuations map must be measured by minimizing the compounding of fluctuations (Equation (13)). Compounding can have several sources, such as temperature [39], vibration [38], and material relaxation by viscoelasticity [18], and of course, constants of time specific to each disturbing phenomenon. Therefore, $\Delta {t\;}_{inter}=0$ will be imposed. However, tests based on temporal model analysis will be performed to verify that the temporal contribution is zero. The Durbin–Watson test will be used for a first order correlation.

### 4.4. Temporal Fluctuations Graph

The intent is to visualize the evolution of fluctuations versus the time of recording, i.e., the index of the map. The problem is that one gets *I* × *J* graphs corresponding to the number of measured positions on individual maps. There are too many curves for one figure. The idea is, therefore, to find six characteristic curves. Six curves corresponding to (*z*_*i*,*j*,*n*_ – *μ*_*i*,*j*_) versus map number corresponding to different ${\hat{\sigma }}_{I,j}$ are retained (Equation (20)). These six curves are mean, median, minimum, maximum, and two percentiles, *Q_5%_* and *Q_95%_*, of the I distribution, respectively noted $\mu_{\hat{\sigma }}$, ${Q50}_{\hat{\sigma }}$, ${min}_{\hat{\sigma }}$, ${max}_{\hat{\sigma }}$,$\;{Q5}_{\hat{\sigma }}$, and ${Q95}_{\hat{\sigma }}$. These curves can also be completed by another standardized representation (Equation (21)),

(20) $${(n,\;z}_{i,j,n}{\;-\;\mu }_{i,j})$$

(21) $$\mathrm{and}\;\left(n,\;\frac{\log_{10}\left|z_{i,j,n}{-\;\mu }_{i,j}\right|}{Sq}\right)$$

with (*i*,*j*) coordinates corresponding to ${\hat{\sigma }}_{i,j}=\left\{\mu_{\hat{\sigma }}\right\}\;$ or ${\hat{\sigma }}_{i,j}=\left\{{Q50}_{\hat{\sigma }}\right\}$ or ${\hat{\sigma }}_{i,j}=\left\{{min}_{\hat{\sigma }}\right\}$ or ${\hat{\sigma }}_{i,j}=\left\{{max}_{\hat{\sigma }}\right\}$ or ${\hat{\sigma }}_{i,j}=\left\{{Q5}_{\hat{\sigma }}\right\}$ or ${\hat{\sigma }}_{i,j}=\left\{{Q95}_{\hat{\sigma }}\right\}$.

### 4.5. Summary of the Methodology

**Figure 4:**
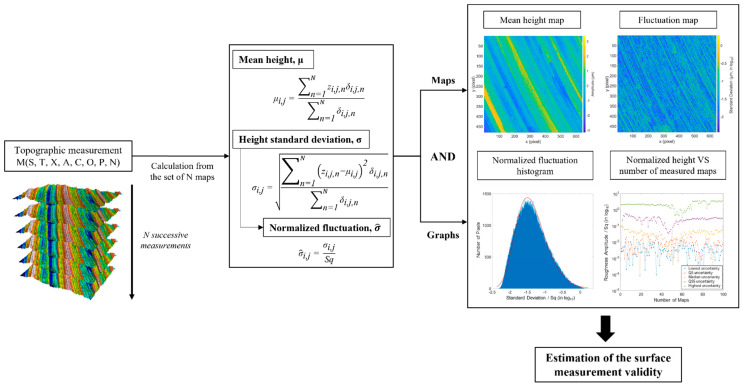
Organigram of the methodology.

Figure 4 is a summary of the calculation on measured heights and generation of graphs and maps to estimate the validity of a surface measurement.

## 5. Results and Discussion

**Figure 5:**
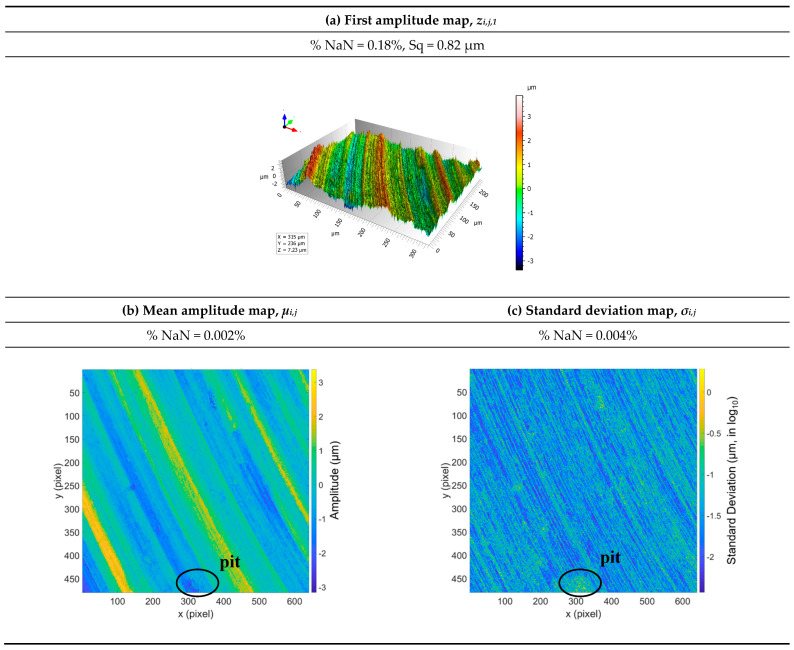
First elementary map of the turned surface (**a**), as well as a mean amplitude map (**b**) and a standard deviation map (**c**) calculated from the set of 100 elementary maps.

The topographic map from the first elementary measurement of the turned surface is shown in Figure 5, along with the mean and standard deviation, i.e., fluctuations, maps for all 100 measurements. For the first elementary measurement, 0.18% of the points *z*_*i*,*j*,1_ (Figure 5a) were non-measured. After 100 measurements, this decreased to 0.002%, so that only two points in one hundred thousand were consistently non-measured all 100 times (Figure 5b). Four positions in one hundred thousand (0.004%) did not have enough measurements (at least two) to calculate a standard deviation (Figure 5c). This averaging operation decreased the number of non-measured points by a factor of 100 on the mean map compared to the first elementary map. There is a stochastic aspect to the occurrence of non-measured points loss at the same positions *(x, y)*.

**Figure 6:**
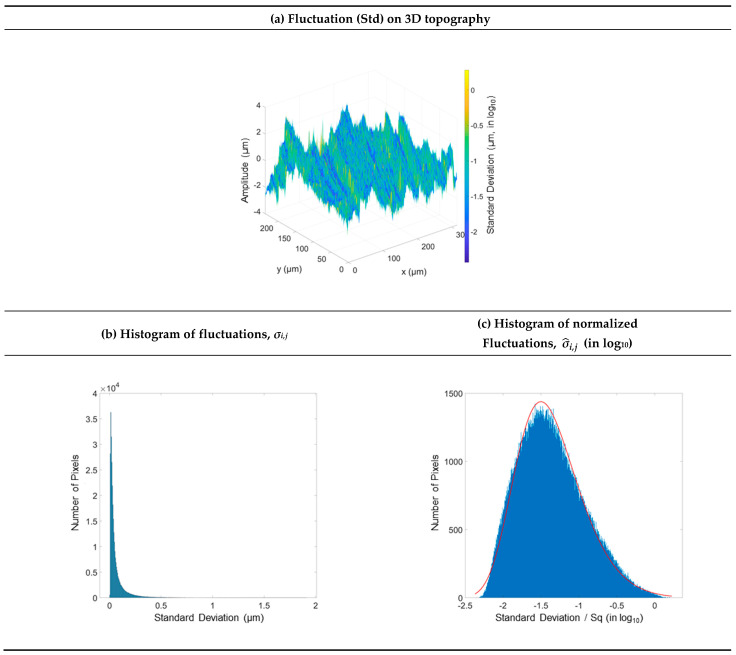
Fluctuations projected on the 3D turned surface topography (**a**), associated histogram of fluctuations σ_i,j_ (**b**), and histogram of normalized fluctuations ${\hat{\sigma }}_{i,j}$ (**c**).

The similarities, intercorrelations, between the mean map *μ*_*i*,*j*_ (Figure 5b) and the standard deviation map *σ*_*i*,*j*__,_ (Figure 5c) are obvious despite the logarithmic scale on the latter. For example, the curves on these maps have the same shape. Another example is the pit centered at the bottom of the maps and circled in Figure 5b,c, where this intercorrelation is also observed. The amplitudes of the features on the standard deviation map can be related to the topographic features but not to the topographic amplitudes. Indeed, the standard deviations do not seem to be related to the amplitude *z*_*i*,*j*,*n*_ because the peaks are not distinguishable from the valleys. Visually, log_10_(*σ_i,j_*) rather suggests that certain topographic shapes, rather than amplitudes, are associated with fluctuations. In order to access this intercorrelation, a tridimensional representation is proposed. The standard deviation map log_10_(*σ_i,j_*), indicating fluctuations, is projected on the 3D height map *μ*_*i*,*j*_ (Figure 6a), so that the apparent topographical origins of the fluctuations can be observed. These fluctuations are concentrated on high surface gradients, i.e., between the ridges and grooves. However, the flatter parts on the tops of the ridges are similar to the flat parts of the valleys, such as: $\log_{10}{\left(\sigma_{i,j}\right)}_{\mathrm{peak}}≃\log_{10}{\left(\sigma_{i,j}\right)}_{\mathrm{valley}}≃\;-2$, i.e., $10^{-2\;}=0.01$ µm of the fluctuation, which corresponds to a mean fluctuation of approximately 1% for an *Sq* of 0.82 µm.

On a histogram of *σ*_*i*,*j*_ (Figure 6b), fluctuations cover a range from 0.004 to 1.9 µm. There is a quasi-vertical rise of *p_σ_* then a slower exponential intensity drop. This trend indicates that fluctuations on real topographies do not always follow Gaussian distributions. This may appear to contradict instrument fluctuations, which are often given as symmetric. Indeed, in the proposed top-down approach, actual fluctuations are calculated on real surfaces, surfaces of practical interest, while in a bottom-up approach, instrument fluctuations measured on wafers are close to a Gaussian or decorrelated white noise. The high skewness of *p_σ_* indicates that convergence of the mean map *μ*_*i*,*j*_ towards the “real” surface *z*_*i*,*j*_ ($\underset{N\to \infty }{lim}\mu_{i,j}\left(N\right){=z}_{i,j}$) is likely to be slow if a maximum global fluctuation of *ϵ* nm on all pixels *μ*_*i*,*j*_ is fixed, such as $\exists N,\;\mathrm{var}\left(\mu_{i,j}\left(N\right)\right)<\;\epsilon^{2},\;\forall i,j$.

Moreover, this high skewness implies a slow convergence towards the Gaussian probability density *μ*_*i*,*j*_ under the central limit theorem with high third-order moments. The Gaussian hypothesis is often used in modeling fluctuation for simplification. The validity of these models can, therefore, be questioned if the fluctuations are no longer negligible and would transform a Gaussian amplitude map into a non-Gaussian map with high heteroscedasticity. Finally, the consequences of fluctuation propagation on computation should be considered. For example, subject to independence between measurements, this rule on the additivity of variances (Equation (22)) is always true and is a relation that does not depend on the probability density function. However, the probability law changes with *n* as does the symmetry of the distribution. The notion of interval can be simplified by reformulating with a probabilistic approach of fluctuations.

(22) $$\overset{□}{\underset{n}{\sum }}\mathrm{var}\left(z_{i,j,n}\right)=n\mathrm{var}\left(z_{i,j}\right)$$

A normalized relation (that does not change the distribution shape) and a variable change in log, i.e.,$\;\log_{10}({\hat{\sigma }}_{i,j})$, are used in order to better express the distribution of the standard deviations or fluctuations (Figure 6c). The histogram becomes more symmetrical but still retains a skewness. Zero indicates that the measurement fluctuation is identical to the RMS roughness ($\sigma /{Sq=1,\;\log}_{10}\left(1\right)=0$).

**Figure 7:**
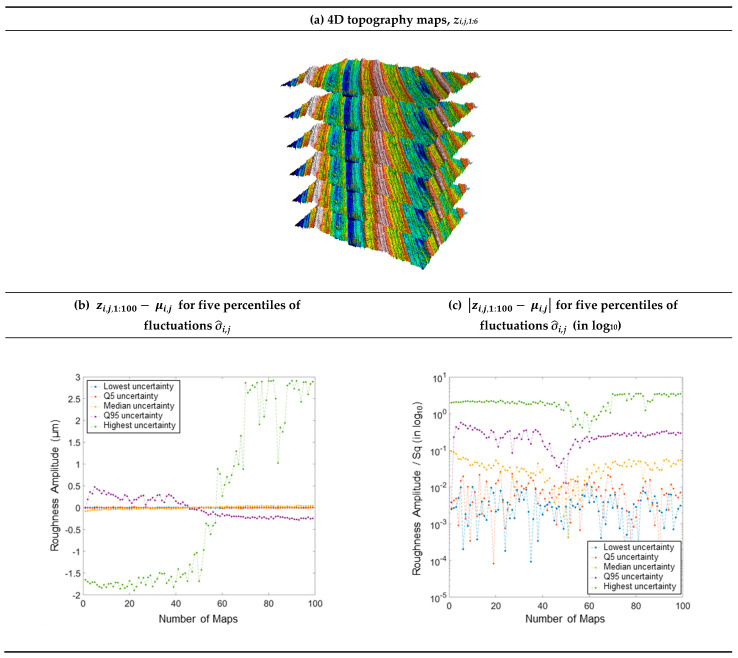
3D topographies of the six elementary maps (**a**), amplitude variation during measurement (**b**), and normalized amplitude variation in log scale (**c**). The lowest, Q_5_, median, Q_95_, and highest fluctuations correspond, respectively, to the minimum, 5% lowest, median, 5% highest, and maximum fluctuations.

Consider the temporal graphs of fluctuations, represented in five instances of height maps (Figure 7a). From Equation (20), Figure 7b is obtained. This figure shows the evolution of the amplitude variation around the mean value during height measurements at the five positions from low to high fluctuation. Indeed, the median is 0.03 µm, the mean is 0.06 µm, and both percentiles, 5^th^ and 95^th^, are respectively 0.008 µm and 0.22 µm. It is noted that the roughness amplitudes increase markedly with the percentiles as previously described. For the median, there is a slight growth of the fluctuations. For the low fluctuations, there is no spatial correlation. There is a temporal correlation of the Q_95_ and maximum curves (Figure 7b). With a log scale (Figure 7c), this phenomenon is more obvious. However, this correlation is inverse; the Q95 curve, initially positive, becomes negative after *n* = 45, whereas the maximum curve, initially negative, becomes positive after approximately *n* = 60 (Figure 7b). Therefore, the inversion of the correlation does not appear at the same number of the map. Moreover, this inverse correlation is surprising because we are looking at the highest fluctuations (maximum) and the 5% highest fluctuations (Q95), so that both curves should involve similarly and not inversely. This does not seem to be implied by a homogeneous height drift of the measuring instrument. This drift is dependent on the fluctuation level. This phenomenon is not explained; some topographical graphs give uncorrelated fluctuation, whereas the general fluctuation is high (see sandblasted surfaces in Table A1 and Table A2). This graph is, therefore, important because it tests the problem of fluctuation autocorrelation.

**Figure 8:**
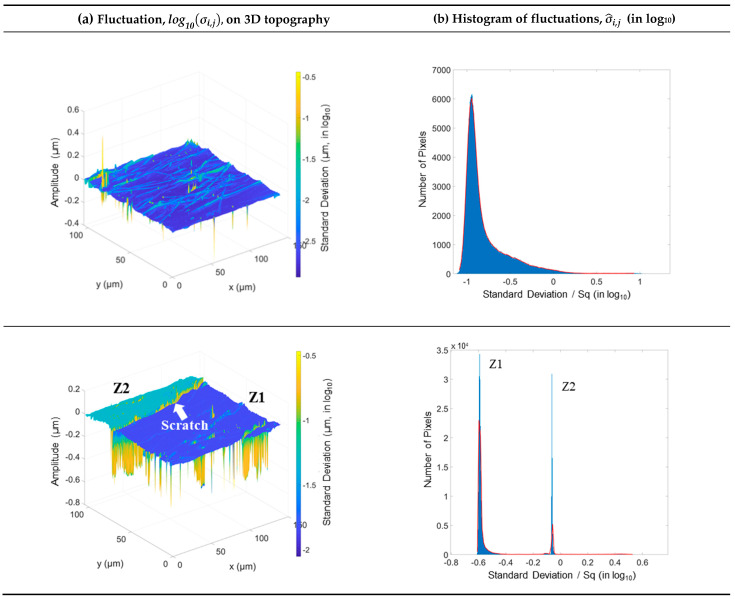
Fluctuations projected on the 3D topographies of the two measured locations of the ophthalmic lens (**a**) and the histogram of the normalized fluctuations (**b**).

For the ophthalmic lens, Figure 8 presents the fluctuations projected on 3D topographic maps (Figure 8a) and the histogram of fluctuations (Figure 8b). More computation graphs on the ophthalmic lens are presented in Table A3 and Table A4. In Figure 8 of the second measured location, the two zones (Z1 and Z2) are clearly distinguishable by their fluctuations and separated by a high amplitude scratch left by the abrasion during the Bayer test. The fluctuation is clearly superior in the low part (Z2) of the scratch with a plateau of approximately $10^{-1.5\;}=0.032$ µm (32 nm) against a plateau of approximately $10^{-1.75\;}=0.018$ µm (18 nm) for Z1. These two fluctuation zones are not due to the surface gradient because they do not appear on the gradient map. This method, therefore, makes it possible to find the fluctuations related to the gradient as well as others. However, the measurements were performed on the same ophthalmic lens at locations relatively close to its center (Figure 3).

This clearly shows the complexity of the interaction between the measuring system and the UTMD fluctuations described by Leach et al. [6]. It seems unreasonable to envisage mathematical or physical models to explain this phenomenon, which has not been encountered in other measurements performed on the biplane lens. Therefore, another top-down approach seems to be responsible for the manifestation of this optical disturbance of interferometer systems on a UTMD surface with complex abrasion mechanisms. This optical artefact could introduce errors in the calculated topographic characterization parameters or even a bias, which would influence the understanding of different tribological phenomena responsible for the damage.

The highest fluctuations correspond to the signatures of the tool/material interaction for the turned surface and to regions with the maximum plastic deformation for the sandblasted surfaces. Finally, for the abraded heterogeneous, multilayer surface, fluctuations are dependent on both abrasion and on the light/sub-layer interactions. The natures of the complex, multiscale surface topographies govern the fluctuation regimes. Topographic regions, which characterize surface functionality or integrity, have the highest fluctuations.

## 6. Conclusions

This pragmatic, probabilistic, top-down method determines position- and situation-specific fluctuations on real, irregular topographies.

Because it does not account for the physical causes of fluctuations, it is not a metrological characterization and cannot be verified by conventional metrological verification methods, such as step heights.

The origin of fluctuations cannot be determined, even if with common sense it is possible that some causes of metrological errors might be supposed (e.g., drift, reference offset).

This method makes it possible to have an estimation of the height fluctuations but does not give information about the causes of these fluctuations. Therefore, it is not possible to know if the fluctuation at a surface point is due to the pixel itself or to an external impact (drift for example).

This method can be applied to any topographic measuring system. Fluctuation maps have distributions of features that are similar to the topographic maps at the same locations, suggesting contributions of some kinds of actual topographic features to augment measurement fluctuations.

Fluctuation distributions can be non-Gaussian and can vary with region on certain surfaces.

In future studies, fluctuation maps will be compared to gradient maps in order to see the influence of surface gradients on the height fluctuations.

## Data Availability

Not applicable.

## Nomenclature

NaN	Non-measured points	
RMS	Root Mean Square	
SWLI	Scanning White Light Interferometry	
UTMD	Uncertainties are Topographically and Material Dependent	
WLI	White Light Interferometry	
*Roughness parameters*		
*Ra*/*Sa*	Arithmetic average roughness (2D/3D)	[µm]
*Rq*/*Sq*	Root mean square roughness (2D/3D)	[µm]
*Measurement information*		
*A*	Measuring instruments	
*C*	Conditions of measurement	
*M*	Set of measured maps (multi-maps)	
*N*	Number of topographic measurements	
*O*	Protocols of measurement	
*P*	Post-processing	
*S*	Surfaces	
*T*	Moments of measurement	
*X*	Locations of measurement	
*Measurement parameters*		
*Δx*	Shift along the x-axis	[µm]
*Δt_intra_*	Necessary time to measure a surface	[s]
*Δt_intra-inter_*	Time of processing and data transfer between two successive measurements	[s]
*Δt_inter_*	Delay time between two successive measurements	[s]
*Δt*	Total time between two successive measurements	[s]
*Δz*	Height difference between two measuring planes	[µm]
*Π*	Form removal polynomial	
*ϕ*	Non-measured point	[NaN]
*d*	Degree of the form removal polynomial	
*D_i_*	Repeatable deviation	
*F_i_*	Fluctuation	
*i*	Coordinates of a surface point along the x-axis	[pixel]
*I*	X size of the measured surface topography	[pixels]
*j*	Coordinates of a surface point along the y-axis	[pixel]
*J*	Y size of the measured surface topography	[pixels]
*k*	Number of the measured scanning plane	
*n*	Number of the measured surface topography	
*r*	Stylus radius	[µm]
*u*	Mesoscopic uncertainties	
*U*	Macroscopic uncertainties	
*v*	Microscopic uncertainties	
*x*	X position in a surface	[µm]
*x_jump_*	X position of the height step on a step surface	[µm]
*y*	Y position in a surface	[µm]
*z*	Height of a surface point	[µm]
*z_x_bottom_*	Height of the bottom plane of a step surface	[µm]
*z_x_top_*	Height of the top plane of a step surface	[µm]
*Z_M_*	Measured height	[µm]
*Z_T_*	Real unknown height	[µm]
*Statistical parameters*		
*μ*	Mean height	[µm]
*σ*	Fluctuation, i.e., standard deviation of the height	[µm]
$\hat{\sigma }$	Normalized fluctuation	
*ϵ*	Maximum global fluctuation	[µm]
*Q*5	5th percentile of $\hat{\sigma }$	
*Q*50	Median of $\hat{\sigma }$	
*Q*95	95th percentile of $\hat{\sigma }$	

## Appendix A

**Table A1:** Sandblasted Surface, 3 Bars.

M(S = {Sandblasting, 3 bar}, A = {ContourGT}, C = {×20}, N = {100})	
Fluctuations,$\;\log_{10}\left(\sigma_{I,j}\right),\;$ on 3D topography	Mean amplitude map, *μ*_*i*,*j*_
% NaN = 15.4%, Sq = 3.7 µm	% NaN = 6.7%

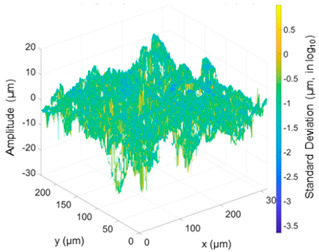
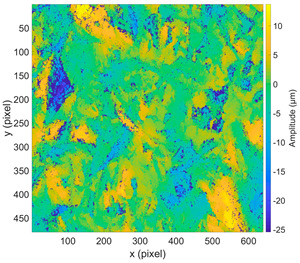
	
Standard deviation map, *σ*_*i*,*j*_	Histogram of fluctuations $\;{\hat{\sigma }}_{i,j}$ (in log_10_)
% NaN = 7.8%	

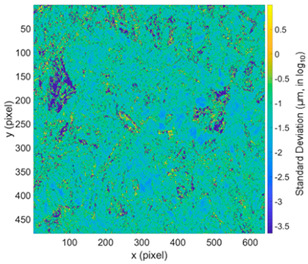
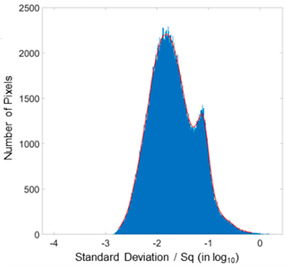
	
$Z_{i,j,1:100}{-\;\mu }_{i,j}$ for five percentiles of fluctuations $\;{\hat{\sigma }}_{i,j}$	$\left|Z_{i,j,1:100}{-\;\mu }_{i,j}\right|$ for five percentiles of fluctuations $\;{\hat{\sigma }}_{i,j}$ (in log_10_)

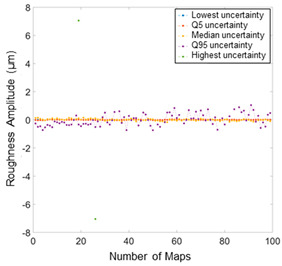
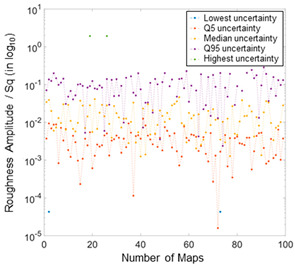
	

**Table A2:** Sandblasted Surface, 6 Bars.

M(S = {Sandblasting, 6 bar}, A = {ContourGT}, C = {×20}, N = {100})	
Fluctuations,$\;\log_{10}\left(\sigma_{I,j}\right),\;$ on 3D topography	Mean amplitude map, *μ*_*i*,*j*_
% NaN = 22.1%, Sq = 5.0 µm	% NaN = 10.7%

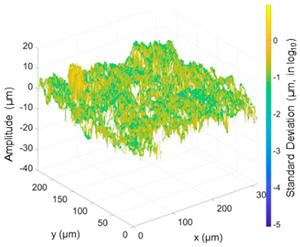
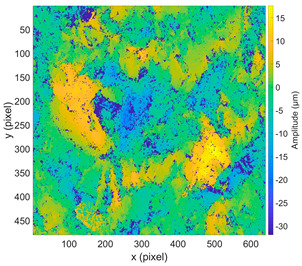
	
Standard deviation map, *σ*_*i*,*j*_	Histogram of fluctuations $\;{\hat{\sigma }}_{i,j}$(in log_10_)
% NaN = 11.4%	

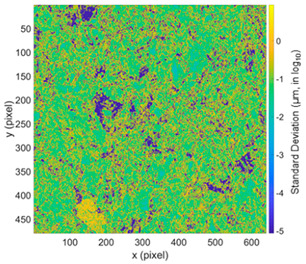
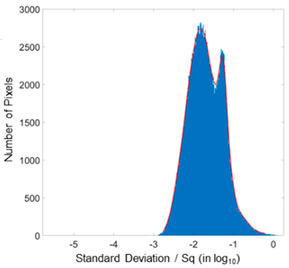
	
$Z_{i,j,1:100}{-\;\mu }_{i,j}$ for five percentiles of fluctuations $\;{\hat{\sigma }}_{i,j}$	$\left|Z_{i,j,1:100}{-\;\mu }_{i,j}\right|$ for five percentiles of fluctuations $\;{\hat{\sigma }}_{i,j}$(in log_10_)

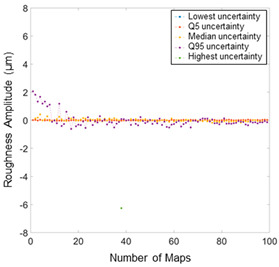
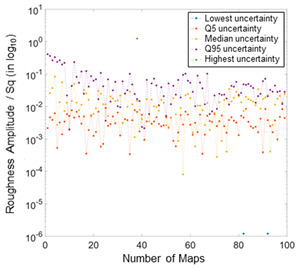
	

**Table A3:** Abraded Ophthalmic Lens (Essilor), Location n°1.

M(S = {Abraded lens}, A = {NewView7300}, {1²}, C = {×50}, N = {100})	
Fluctuations, $\log_{10}\left(\sigma_{i,j}\right),\;$ on 3D topography	Mean amplitude map, *μ*_*i*,*j*_
% NaN = 0.14%, Sq = 16.4 nm	% NaN = 0.0006%

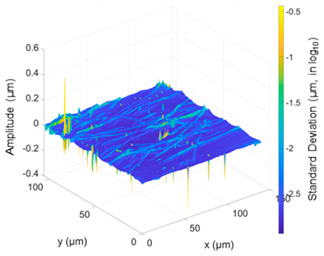
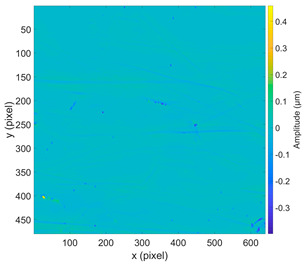
	
Standard deviation map, *σ*_*i*,*j*_	Histogram of fluctuations ${\hat{\sigma }}_{i,j}$(in log_10_)
% NaN = 0.001%	

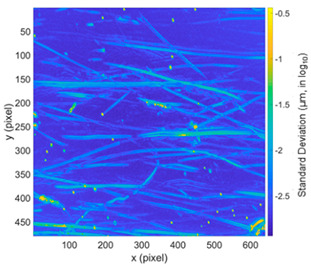
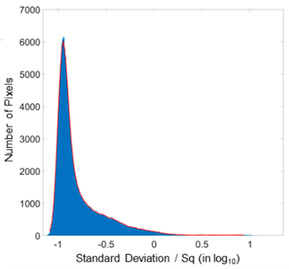
	
$Z_{i,j,1:100}{-\;\mu }_{i,j}$ for five percentiles of fluctuations ${\hat{\sigma }}_{i,j}$	$\left|Z_{i,j,1:100}{-\;\mu }_{i,j}\right|$ for five percentiles of fluctuations ${\hat{\sigma }}_{i,j}$(in log_10_)

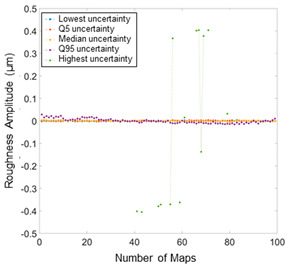
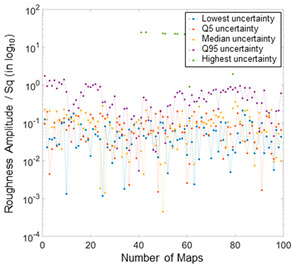
	

**Table A4:** Abraded Ophthalmic Lens (Essilor), Location n°2.

M(S = {Abraded lens}, A = {NewView7300}, {2²}, C = {×50}, N = {100})	
Fluctuations, $\log_{10}\left(\sigma_{I,j}\right),\;$ on 3D topography	Mean amplitude map, *μ*_*i*,*j*_
% NaN = 0.10%, Sq = 56.0 nm	% NaN = 0%

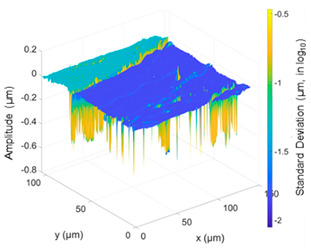
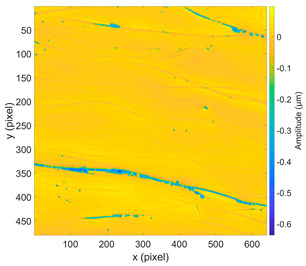
	
Standard deviation map, *σ*_*i*,*j*_	Histogram of fluctuations ${\hat{\sigma }}_{i,j}$(in log_10_)
% NaN = 0.00031%	

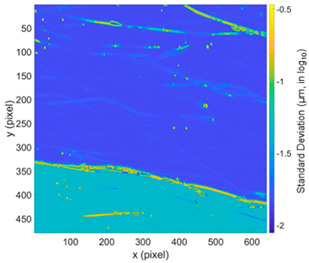
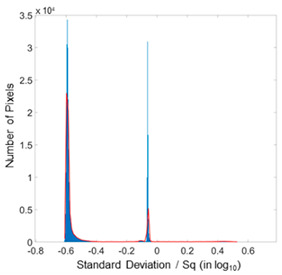
	
$Z_{i,j,1:100}{-\;\mu }_{i,j}$ for five percentiles of fluctuations ${\hat{\sigma }}_{i,j}$	$\left|Z_{i,j,1:100}{-\;\mu }_{i,j}\right|$ for five percentiles of fluctuations ${\hat{\sigma }}_{i,j}$(in log_10_)
	
